# Spirometry in Central Asian Lowlanders and Highlanders, a Population Based Study

**DOI:** 10.3389/fmed.2019.00308

**Published:** 2020-01-10

**Authors:** Stefanie Ulrich, Michael Furian, Bermet Estebesova, Nurgul Toktogulova, Gulnara Beishekeeva, Silvia Ulrich, Peter G. J. Burney, Talant M. Sooronbaev, Konrad E. Bloch

**Affiliations:** ^1^Department of Respiratory Medicine, University Hospital Zurich, Zurich, Switzerland; ^2^Department of Respiratory Medicine, National Center for Cardiology and Internal Medicine, Bishkek, Kyrgyzstan; ^3^Therapy 1 Department, Medical Faculty, Kyrgyz-Russian Slavic University, Bishkek, Kyrgyzstan; ^4^Population Health and Occupational Disease, National Heart and Lung Institute & MRC-PHE Centre for Environment and Health, Imperial College London, London, United Kingdom

**Keywords:** spirometry, altitude, lung function, Central Asian, highlanders

## Abstract

**Introduction:** The purpose of the study was to establish spirometric reference values for a Central Asian population of highlanders and lowlanders.

**Methods:** Spirometries from a population-based cross-sectional study performed in 2013 in rural areas of Kyrgyzstan were analyzed. Using multivariable linear regression, Global Lung Function Initiative (GLI) equations were fitted separately for men and women, and altitude of residence (700–800 m, 1,900–2,800 m) to data from healthy, never-smoking Kyrgyz adults. The general GLI equation was applied:

$$\begin{array}{l} \text{Predicted value}={\text{e}}^{{\text{a}}_{0}+{\text{a}}_{1}\times \text{ ln}\left(\text{Height}\right)+{\text{a}}_{2}\times \text{ ln}\left(\text{Age}\right)+{\text{b}}_{1}\times \text{ ln}\left(\frac{\text{Age}}{100}\right)+{\text{b}}_{2}\times \text{ ln}{\left(\frac{\text{Age}}{100}\right)}^{2}+{\text{b}}_{3}\times \text{ ln}{\left(\frac{\text{Age}}{100}\right)}^{3}}\\ {\;}^{+{\text{b}}_{4}\times \text{ ln}{\left(\frac{\text{Age}}{100}\right)}^{4}+{\text{b}}_{5}\times \text{ ln}{\left(\frac{\text{Age}}{100}\right)}^{5}} \end{array}$$

**Results:** Of 2,784 screened Kyrgyz, 448 healthy, non-smoking highlanders (379 females) and 505 lowlanders (368 females), aged 18–91 years, were included. Predicted FVC in Kyrgyz fit best with GLI “North-East Asians,” predicted FEV_1_ fit best with GLI “Other/Mixed.” Predicted FEV_1_/FVC was lower than that of all GLI categories. Age- and sex-adjusted mean FVC and FEV_1_ were higher in highlanders (+0.138l, +0.132l) than in lowlanders (*P* < 0.001, all comparisons), but FEV_1_/FVC was similar.

**Conclusion:** We established prediction equations for an adult Central Asian population indicating that FVC is similar to GLI “North-East Asian” and FEV_1_/FVC is lower than in all other GLI population categories, consistent with a relatively smaller airway caliber. Central Asian highlanders have significantly greater dynamic lung volumes compared to lowlanders, which may be due to environmental and various other effects.

## Introduction

Ethnic differences in pulmonary function have been frequently reported and spirometry reference equations for various populations and ethnic groups have been published in the past (1–7). For individual diagnostic evaluation and epidemiological investigations, well-based reference values, derived from healthy individuals of the local population, are essential (8).

Little research has been published in the English literature on lung function among Central Asian adults. Around 70 million people inhabit this area (9), applying the most common definition of Central Asia. The Kyrgyz Republic, as an example of Central Asian states, is home to more than six million inhabitants (10) and around 41% of the population live higher than 1,000 m above sea level (11). A complex history of colonization with multiple waves of migration from East and West has resulted in a heterogeneous mix of races and ethnicities living in Central Asia (12–14). While spirometry reference values calculated from measurements in 1,044 Kyrgyz male miners (15) and from Kazak children (16) have been reported, spirometric reference data of a large Central Asian population sample of healthy, non-smoking adults—especially those of women—are lacking. As recommended by the American Thoracic Society (ATS) and the European Respiratory Society (ERS) (17, 18), validating published reference equations for a healthy sample of the local community would be desirable. The Global Lung Function Initiative (GLI) equations are the most recent published prediction equations for spirometry based on 97'759 spirometries of healthy individuals from 72 centers in 33 countries (19). Depending on age, height, sex and ethnicity, the forced vital capacity (FVC), the forced expiratory volume in one second (FEV_1_) and the FEV_1_/FVC ratio have been predicted for five ethnic groups that do not include Central Asians.

The objective of the present research was therefore to generate prediction equations for spirometry for Central Asian adults using the GLI equations and data collected in a random population sample investigated within the scope of the burden of lung disease survey (BOLD) (20). Since previous studies (21, 22) have provided conflicting evidence on the effect of altitude of residence on lung volumes, an additional aim of the current study was to compare lung function of highlanders with that of lowlanders.

## Methods

### Study Design and Setting

Data of this cross sectional study of a Central Asian population sample were collected within the scope of the Burden of Obstructive Lung Disease (BOLD) survey that has been designed to evaluate the prevalence and burden of chronic obstructive pulmonary disease (COPD). The measurements took place in summer 2013 in two districts (oblasts) of the Kyrgyz Republic, namely the Chui (altitude 700–800 m, mean barometric pressure 695 mmHg) and Naryn (altitude 1,900–2,800 m, mean barometric pressure 571 mmHg). Detailed methods have been reported (20).

### Participants

Participants were invited from a random sample of the inhabitants of the Chui and Naryn area. These districts represented 20% of the total population of Kyrgyzstan (23). The randomization and recruitment was performed following the BOLD protocol (20) in a multi-stage procedure. At first, 10 settlements were randomly sampled from a list of 61 settlements in Naryn and 239 settlements in Chui, respectively. The elevation for each settlement was recorded. Secondly, 50 households in the selected settlements were randomly selected. Thirdly, all subjects aged older than 18 years were invited to take part in the survey.

In the Chui area, 822'581 inhabitants (404'655 men and 417'926 women) were resident in 2012 (24) at altitudes of 700–800 m and therefore termed lowlanders. This region has a continental climate with hot summers and moderately cold winters. At the time of the survey, major industries were an ore-mining factory, a concrete & slate factory, as well as a glass factory. In the Naryn area, 264'947 inhabitants (134'338 men and 130'609 women) resided at altitudes between 1,900 and 2,800 m and were designated highlanders. They were exposed not only to hypobaric hypoxia but also to rather tough weather conditions and indoor air pollution due to the use of biomass for heating and cooking (25). Most inhabitants of both areas were living in rural conditions and worked in the agricultural sector.

To obtain a healthy population sample, the following exclusion criteria were applied according to GLI (4): smokers and ex-smokers, known respiratory disease or symptoms (cough, wheezing, phlegm, and shortness of breath), relevant co-morbidity (heart disease, heart failure, diabetes, tuberculosis, and stroke).

### Assessments

The data were collected during home visits. The BOLD core questionnaire (20) was used to obtain information about medical history, respiratory disease, respiratory symptoms, smoking habits, co-morbidities, and ethnicity. Height and weight were measured and recorded together with age and sex and the body mass index (BMI) was calculated.

Lung function was assessed using an ultrasound transit time flow meter (EasyOneTN, NDD, Zurich, Switzerland). High quality spirometry maneuvers were ensured with the protocol described by BOLD (20) (see online Supplement for additional information). A valid spirometry was defined as a measurement before inhalation of a short acting bronchodilator fulfilling quality criteria according to ATS.

### Statistics

Analysis was performed according to the per protocol principle with all available valid data. Detailed descriptions of all calculations can be found in the online Supplement. According to the GLI, reference equations were fitted to measured FVC, FEV_1_, FEV_1_/FVC, and peak expiratory flow (PEF) values, respectively, using multivariable linear regression analyses. Predicted values were computed as:

$$\begin{array}{l} \text{Predicted value }\left(\text{in liters}\right)={\text{e}}^{{\text{a}}_{0}+{\text{a}}_{1}\times \text{ ln}\left(\text{Height}\right)+{\text{a}}_{2}\times \text{ ln}\left(\text{Age}\right)}\\ {\;}^{+{\text{b}}_{1}\times \text{ ln}\left(\frac{\text{Age}}{100}\right)+{\text{b}}_{2}\times \text{ ln}{\left(\frac{\text{Age}}{100}\right)}^{2}+{\text{b}}_{3}}\\ {\;}^{\times \text{ ln}{\left(\frac{\text{Age}}{100}\right)}^{3}+\;{\text{b}}_{4}\times \text{ ln}{\left(\frac{\text{Age}}{100}\right)}^{4}+\;{\text{b}}_{5}\times \text{ ln}{\left(\frac{\text{Age}}{100}\right)}^{5}} \end{array}$$

The mean (M), the coefficient of variation (S) and the skewness (L) were predicted for each sex depending on age and height. To obtain a continuous smooth fit over the age range, splines for M (Mspline), L (Lspline), and S (Sspline) were introduced using the Box-Cox-Cole-Green distribution (26, 27) according to the GLI (4).

The predicted mean (M), lower limit of normal (LLN) and percent predicted (PP) were calculated:

M = predicted spirometry value

$$\begin{array}{lll} \text{LLN} & = & \;{\text{e}}^{\frac{\text{ln}\left(1-1.644\times \text{L}\times \text{S}\right)}{\text{L}}+\text{ lnM}}\\ \text{PP} & = & \;\frac{\text{measured}}{\text{M}}\times 100 \end{array}$$

For the PEF, L was assumed to be zero and the LLN was calculated with the standard deviation (SD).

$$\begin{array}{l} \text{LLN}=\text{M}-1.644\times \text{SD} \end{array}$$

Difference between highlanders and lowlanders were evaluated by including living altitude split into two categories, highlanders (1,900–2,800 m) and lowlanders (700–800 m) into the regression analyses.

Statistics were performed with Stata/SE V15.1 software (Stata Corp., Texas, USA). Additional details are provided in an online Supplement.

## Results

**Figure 1:**
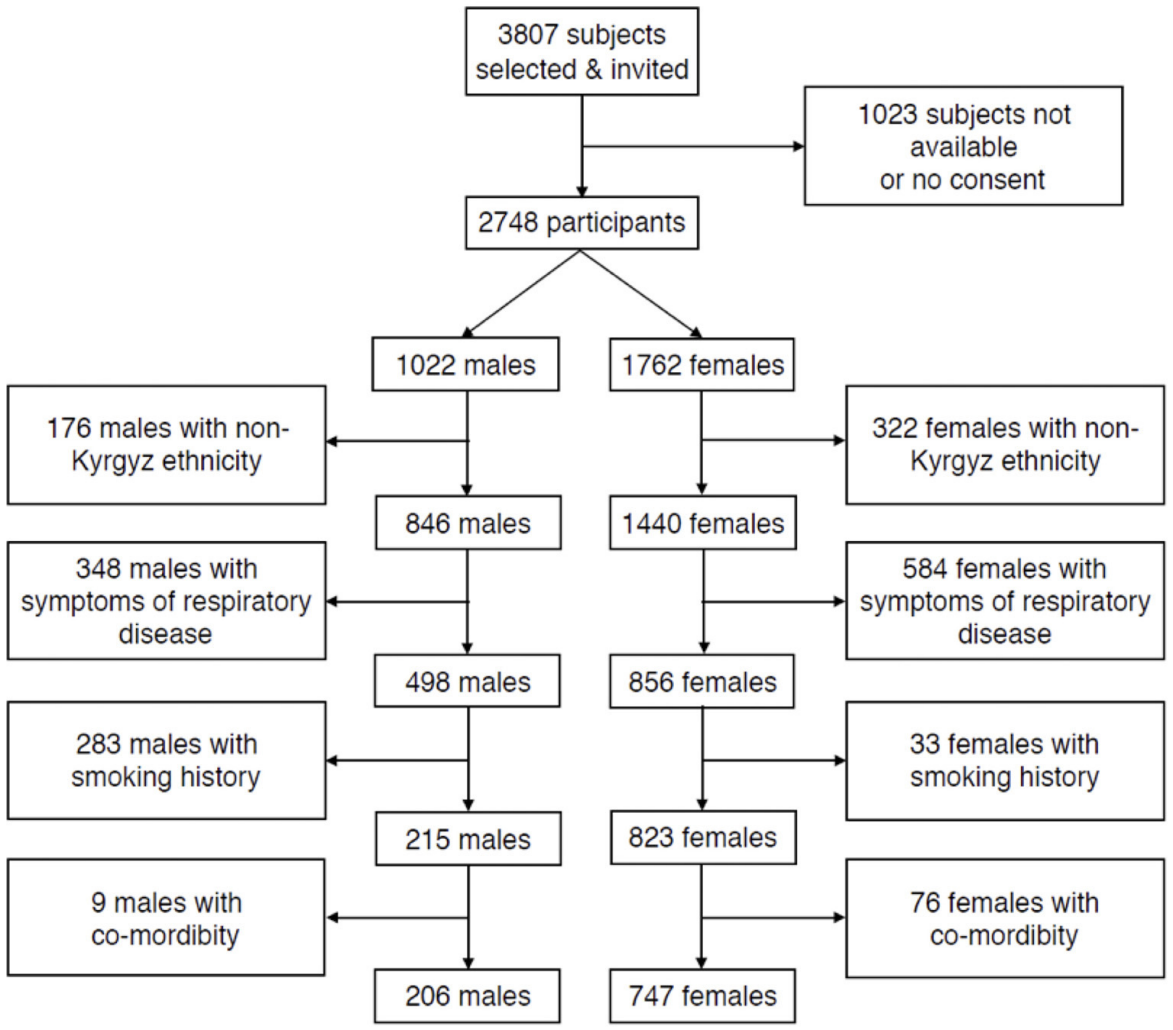
Subject flow.

In total, 3,804 subjects were invited, of whom 2,784 participated (Figure 1). Once we applied the exclusion criteria selecting healthy individuals, 206 males (69 lowlanders; 137 highlanders) and 747 females (379 lowlanders; 368 highlanders), aged 18–91 years, were included into the final analyses. Separate flow diagrams, for highlanders and lowlanders are shown in Supplementary Figures 1, 2. The main reasons of exclusion were symptoms of respiratory diseases (*n* = 891), smoking history (*n* = 667), and non-Kyrgyz ethnicity (*n* = 416).

The lowlanders and highlanders show comparable baseline characteristics for age and body-mass-index (BMI) (Table 1). The height of highlanders of both sexes and the weight of female highlanders were significantly lower than corresponding values of lowlanders. The distributions of age, height and weight are displayed in Supplementary Figure 3.

**Table 1:** Participant characteristics.

	**All**		**Lowlanders (730 m)**		**Highlanders (1,900–2,800 m)**	
	**Men**	**Women**	**Men**	**Women**	**Men**	**Women**
Number (*n*)	206	747	69	379	137	368
Age (years)	37 (25;52)	42 (33;51)	33 (23;47)	42 (32;50)	40 (26;53)	42 (34;52)
Height (cm)	170 (166;174)	157 (153;161)	171 (167;176)	157 (154;162)	169^*^ (166;173)	156^*^ (152;160)
Weight (kg)	67 (61;76)	63 (55;73)	69 (62;80)	64 (56;75)	67 (61;75)	63^*^ (54;71)
BMI (kg/m^2)^	23.2 (21.0;26.6)	25.9 (22.5;29.1)	23.1 (21.5;27.4)	26.4 (22.5;29.9)	23.2 (21.0;26.4)	25.5 (22.5;28.5)

*Median (lower quartile; upper quartile)*.

* *p < 0.05 lowlanders vs. highlanders*.

**Figure 2:**
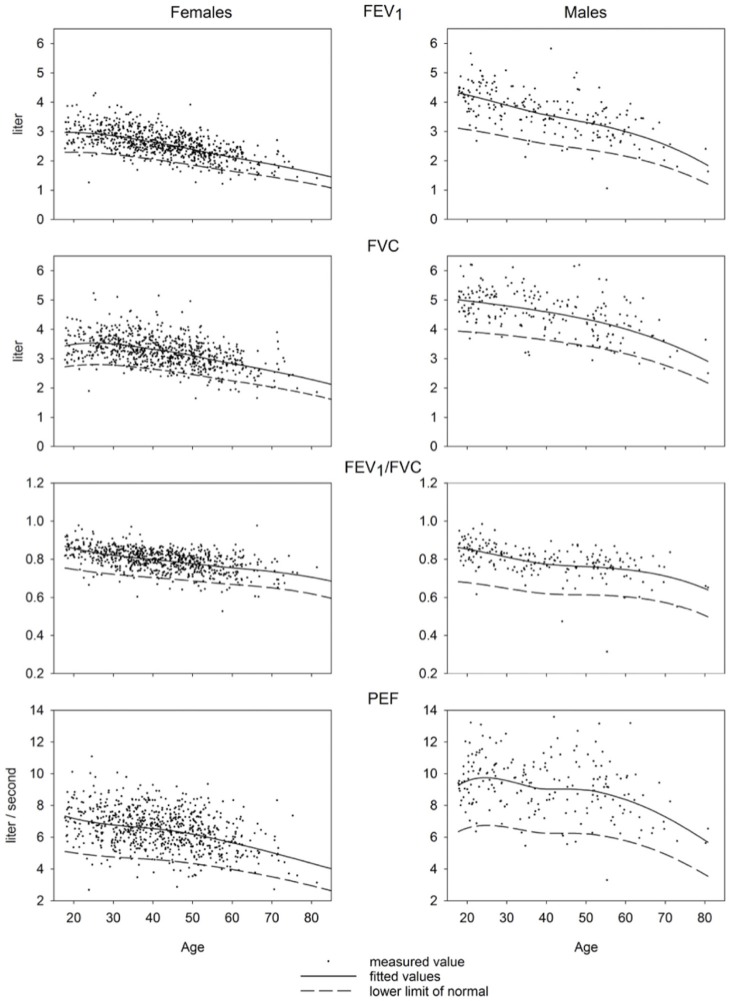
Predicted values for FEV_1_, FVC, FEV_1_/FVC, and PEF.

Measured values of spirometric variables along with predicted means and LLN as a function of age are displayed in Figure 2. Equations and values of coefficients for the calculations of predicted values are presented in Supplementary Tables 1–4.

**Figure 3:**
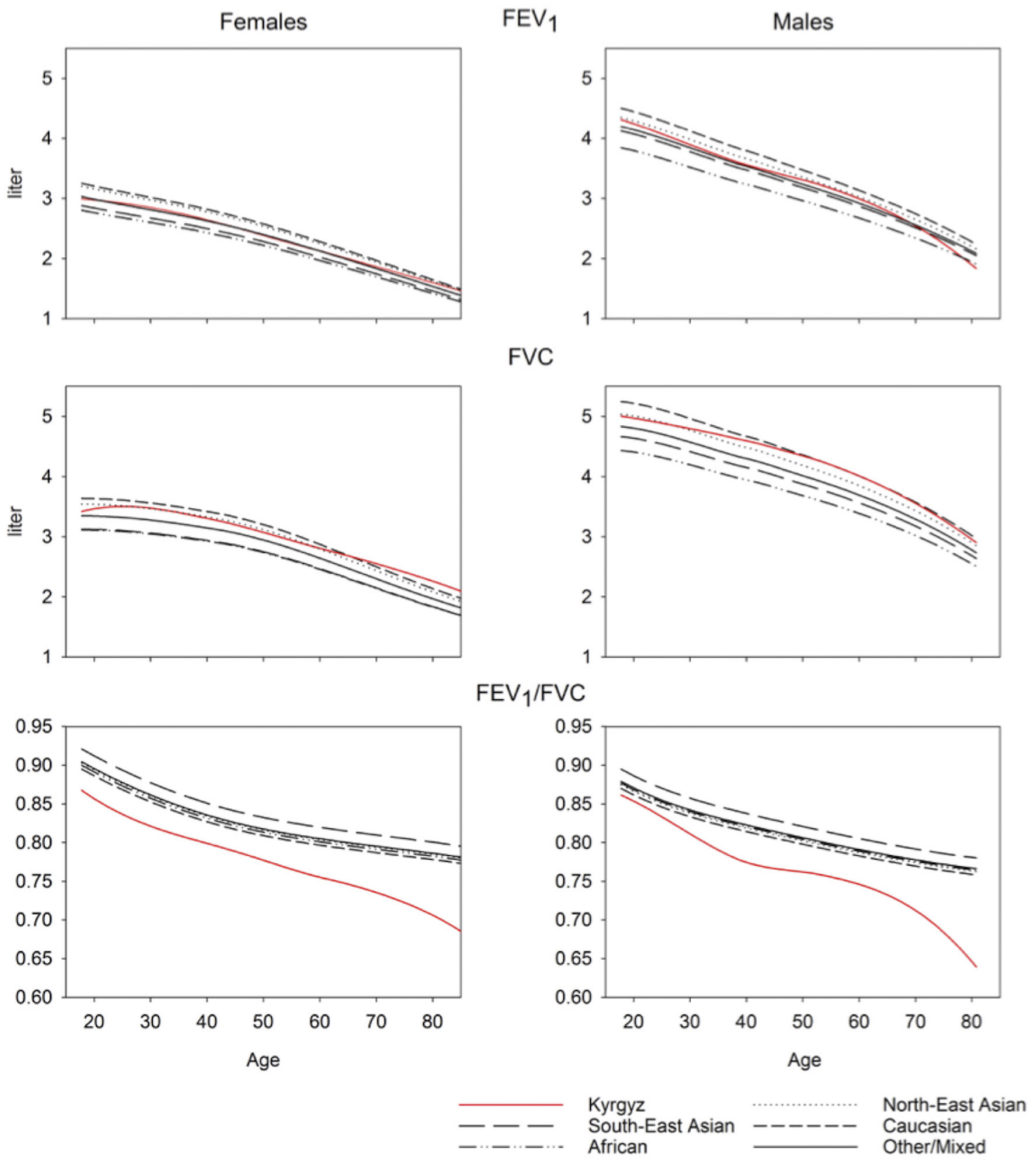
Ethnicity comparison.

A comparison of the equations for Central Asia with that of other ethnic groups described by GLI equations is shown in Figure 3. Predicted values for FVC in Central Asia are similar to those of North-East Asians, predicted values for FEV_1_ are similar to those of Other/Mixed, the FEV_1_/FVC ratio is lower than that of all other ethnic groups described in the GLI reference set. To illustrate the difference between spirometric indices predicted for various groups, we computed differences between indices predicted for specific groups, i.e., Kyrgyz lowlanders, Kyrgyz highlanders and other GLI categories and subtracted corresponding values for all Kyrgyz. These differences are shown in Table 2 for males and Table 3 for females. Separate results are presented for individuals up to 40 years and >40 years of age because there was an age dependent trend in the deviation between the groups (Figure 3).

**Table 2:** Difference in spirometric indices predicted for various groups minus corresponding values predicted for all Kyrgyz men.

**FEV_1_**	**Age** **≤** **40 years**		**Age** **>** **40 years**	
	**Delta^◦^ (%)**	***P***	**Delta^◦^ (%)**	***P*–value**
All Kyrgyz men (= reference group)	0	n.a.	0	n.a.
Kyrgyz lowlanders	−1.5 (−1.5 to −1.4)	<0.001	−1.8 (−1.9 to −1.7)	<0.001
Kyrgyz highlanders	0.9 (0.9 to 1.0)	<0.001	0.7 (0.6 to 0.7)	<0.001
Caucasians*	5.2 (5.0 to 5.4)	<0.001	5.5 (5.0 to 6.0)	<0.001
SE Asians*	−3.5 (−3.8 to −3.3)	<0.001	−3.4 (−4.0 to −2.9)	<0.001
NE Asians*	1.8 (1.6 to 2.0)	<0.001	2.1 (1.5 to 2.6)	<0.001
Africans*	−11.1(−11.5 to−10.8)	<0.001	−11.1(−11.9 to−10.4)	<0.001
Other/Mixed*	−1.8 (−2.0 to −1.6)	<0.001	−1.6 (−2.1 to −1.1)	<0.001
**FVC**				
All Kyrgyz men	0	n.a.	0	n.a.
Kyrgyz lowlanders	0.1 (−0.1 to 0.1)	<0.001	0.1 (−0.1 to 0.1)	<0.001
Kyrgyz highlanders	−0.1 (−0.1 to −0.1)	<0.001	−0.0 (−0.0 to −0.0)	<0.001
Caucasians*	3.6 (3.2 to 3.9)	<0.001	0.4 (0.1 to 0.8)	0.008
SE Asians*	−8.5 (−8.9 to −8.1)	<0.001	−12.2(−12.7 to−11.6)	<0.001
NE Asians*	−0.4 (−0.8 to −0.1)	0.009	−3.7 (−4.1 to −3.4)	<0.001
Africans*	−14.2(−14.7 to−13.7)	<0.001	−18.1(−18.7 to−17.4)	<0.001
Other/Mixed*	−4.8 (−5.1 to −4.4)	<0.001	−8.2 (−8.7 to −7.8)	<0.001
**FEV**_**1**_**/FVC**				
All Kyrgyz men	0	n.a.	0	n.a.
Kyrgyz lowlanders	−1.6 (−1.6 to −1.5)	<0.001	−1.9 (−1.9 to −1.8)	<0.001
Kyrgyz highlanders	1.0 (0.9 to 1.0)	<0.001	0.7 (0.6 to 0.7)	<0.001
Caucasians*	2.3 (2.0 to 2.5)	<0.001	5.2 (4.8 to 5.6)	<0.001
SE Asians*	5.0 (4.7 to 5.3)	<0.001	7.8 (7.4 to 8.2)	<0.001
NE Asians*	2.8 (2.5 to 3.1)	<0.001	5.7 (5.3 to 6.1)	<0.001
Africans*	3.0 (2.7 to 3.3)	<0.001	6.0 (5.6 to 6.3)	<0.001
Other/Mixed*	3.3 (3.0 to 3.6)	<0.001	6.2 (5.8 to 6.6)	<0.001
**PEF**				
All Kyrgyz men	0	n.a.	0	n.a.
Kyrgyz lowlanders	−2.0 (−2.0 to −1.9)	<0.001	−2.3 (−2.4 to −2.2)	<0.001
Kyrgyz highlanders	1.2 (1.1 to 1.2)	<0.001	0.9 (0.8 to 1.0)	<0.001

*FEV_1_, forced expiratory volume in 1s; FVC, forced vital capacity; PEF, peak expiratory flow; n.a, not available*.

◦ Delta (%) corresponds to the difference in percent predicted by equations for Kyrgyz highlanders or lowlanders or one of the GLI categories (*) for minus the corresponding value predicted by equations for all Kyrgyz men.

**Table 3:** Difference in spirometric indices predicted by equations for various groups minus corresponding indices predicted for all Kyrgyz women.

**FEV_**1**_**	**Age** **≤** **40 years**		**Age** **>** **40 years**	
	**Delta^**◦**^ (%)**	***P***	**Delta^**◦**^ (%)**	***P***
All Kyrgyz	0	n.a.	0	n.a.
Kyrgyz lowlanders	−2.5 (−2.6 to −2.5)	<0.001	−2.6 (−2.7 to −2.5)	<0.001
Kyrgyz highlanders	2.6 (2.6 to 2.7)	<0.001	2.6 (2.5 to 2.6)	<0.001
Caucasians*	5.9 (5.8 to 6.1)	<0.001	6.7 (6.4 to 7.0)	<0.001
SE Asians*	−6.2 (−6.5 to 6.0)	<0.001	−5.4 (−5.7 to −5.1)	<0.001
NE Asians*	4.5 (4.3 to 4.7)	<0.001	5.3 (5.0 to 5.5)	<0.001
Africans*	−9.2 (−9.5 to −9.0)	<0.001	−8.3 (−8.7 to −8.1)	<0.001
Other/Mixed*	−1.0 (−1.2 to 0.8)	<0.001	−0.2 (−0.5 to 0.0)	0.071
**FVC**				
All Kyrgyz	0	n.a.	0	n.a.
Kyrgyz lowlanders	−2.3 (−2.4 to −2.3)	<0.001	−2.4 (−2.5 to −2.4)	<0.001
Kyrgyz highlanders	2.4 (2.4 to 2.5)	<0.001	2.4 (2.3 to 2.4)	<0.001
Caucasians*	2.9 (2.7 to 3.1)	<0.001	3.0 (2.7 to 3.2)	<0.001
SE Asians*	−13.1(−13.4 to−12.8)	<0.001	−13.1(−13.4 to−12.7)	<0.001
NE Asians*	0.3 (0.1 to 0.5)	0.002	0.4 (0.1 to 0.6)	0.013
Africans*	−13.5(−13.8 to−13.3)	<0.001	−13.5(−13.9 to−13.1)	<0.001
Other/Mixed*	−5.6 (−5.8 to −5.4)	<0.001	−5.5 (−5.9 to −5.2)	<0.001
**FEV**_**1**_**/FVC**				
All Kyrgyz	0	n.a.	0	n.a.
Kyrgyz lowlanders	−0.2 (−0.2 to −0.2)	<0.001	−0.2 (−0.2 to −0.2)	<0.001
Kyrgyz highlanders	0.2 (0.2 to 0.2)	<0.001	0.2 (0.2 to 0.2)	<0.001
Caucasians*	3.5 (3.5 to 3.6)	<0.001	4.2 (4.1 to 4.4)	<0.001
SE Asians*	6.3 (6.2 to 6.3)	<0.001	6.9 (6.8 to 7.1)	<0.001
NE Asians*	4.4 (4.4 to 4.4)	<0.001	5.1 (5.0 to 5.2)	<0.001
Africans*	4.1 (4.0 to 4.1)	<0.001	4.8 (4.6 to 4.9)	<0.001
Other/Mixed*	4.6 (4.5 to 4.6)	<0.001	5.3 (5.1 to 5.4)	<0.001
**PEF**				
All Kyrgyz	0	n.a.	0	n.a.
Kyrgyz lowlanders	−3.5 (−3.6 to −3.4)	<0.001	−3.6 (−3.7 to −3.6)	<0.001
Kyrgyz highlanders	3.7 (3.6 to 3.7)	<0.001	3.5 (3.4 to 3.6)	<0.001

*FEV_1_, forced expiratory volume in 1s; FVC, forced vital capacity; PEF, peak expiratory flow; n.a, not available*.

◦ Delta (%) corresponds to the difference in percent predicted by equations for Kyrgyz highlanders or lowlanders or one of the GLI categories (*) minus the corresponding value predicted by equations for all Kyrgyz women.

**Figure 4:**
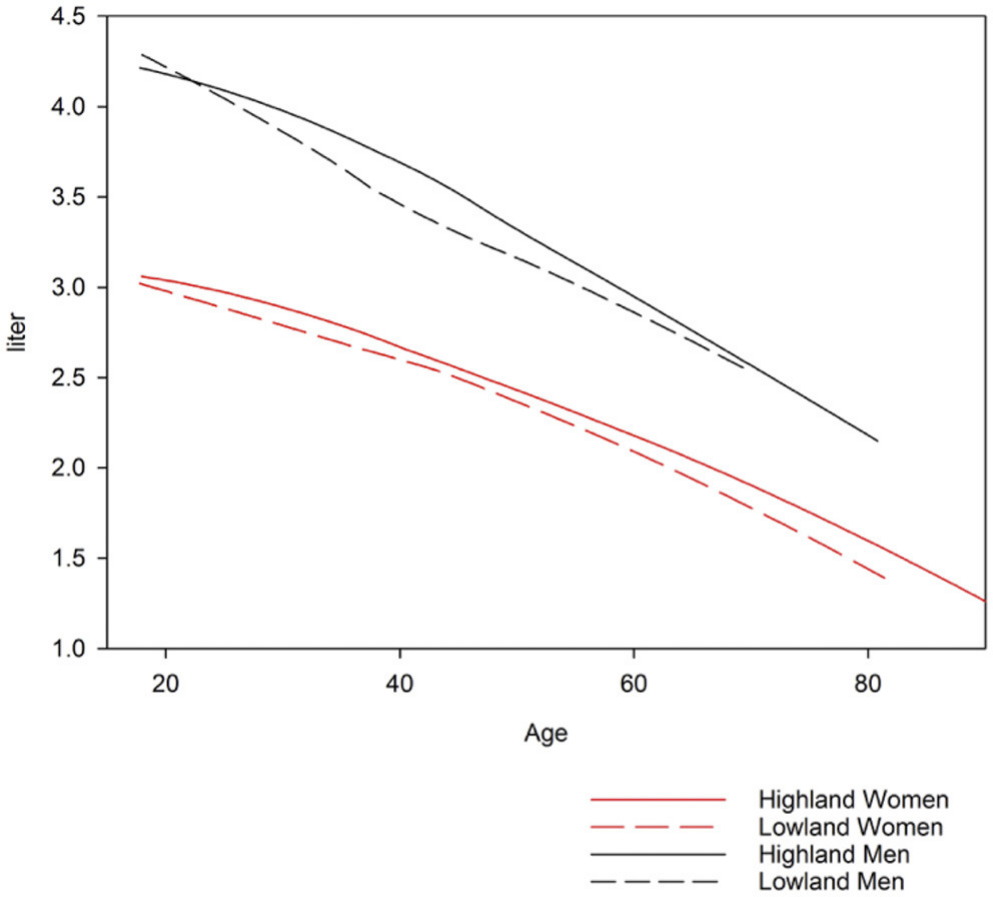
Forced expiratory volume in one second for lowlanders and highlanders.

Highlanders compared to lowlanders have higher FEV_1_ (mean difference: 0.132 L; *p*-value < 0.001), FVC (0.138 L; < 0.001), FEV_1_/FVC (0.006, 0.108) and PEF (0.460 L/s; < 0.001) corrected for age, height and sex (Figure 4, Tables 2, 3).

## Discussion

To our knowledge, we report the first spirometry reference values for a Central Asian population applying the GLI equations. The main findings were an FVC comparable with the GLI “North-East Asian” category, an FEV_1_ comparable with the GLI “Other/Mixed” category and an FEV_1_/FVC lower than all other ethnicities described by GLI. In highlanders, values for FEV_1_, FVC and PEF were higher compared to lowlanders.

It seems to be plausible that Central Asians resemble North-East Asians and Caucasians in lung function indices as they geographically populate an area historically influenced by both of these groups as supported by mitochondrial DNA analyses (14). The low FEV_1_/FVC found in Kyrgyz men and women is intriguing, as this ratio is used for definition of COPD and seems to be higher in large populations of other regions of the world (4). Applying a defined threshold established in other ethnicities, a higher percentage of Central Asian subjects would be diagnosed with airflow obstruction. In a sample of Kyrgyz male miners a systematic overestimation of FEV_1_/FVC by the GLI and Mongolian (28) equations (15) have been reported. Results are not fully comparable to our study as the sample of miners was not representative for the general adult population. Additionally, no inference about the influence of altitude could be made as subjects worked, resided and performed spirometry at different altitudes (3,800, 1,600, 750 m, respectively) and no information on the duration of stay at each place was provided. As suggested by observations in previous large population samples in South-East Asia (4) and the Middle East (29), genetic and environmental factors including exposure to altitude or excessive air pollution (30) and low socioeconomic status (31) may be responsible for differences in FEV_1_/FVC between the cited groups. Other reasons for our findings could be a low effort in FEV_1_ performance, even though we applied the spirometry quality management procedure of BOLD or that Kyrgyz have a different perception of respiratory symptoms resulting in inclusion of certain participants with airway disease.

We performed a comparison among subjects with the same ethnic background, living on 700–800 m and 1,900–2,800 m. We found a significantly shorter body size of male and female highlanders compared to lowlanders by 1–2 cm (Table 1). It has been proposed that due to hypoxia, body growth *in utero*, and early childhood is slowed down and lung growth is stimulated (32). Therefore, especially among children, these differences are more pronounced. In the Andean highlands a retardation of growth of 1–4 cm was measured (33). Consistent with this, Weitz et al. observed a lower stature in first generation Han Chinese living in the Himalayas at altitude (above 3,200 m) in comparison to Han Chinese living at lower altitudes (34) suggesting that environmental factors were important determinants of growth. However, the influence of nutrition difference and selection bias due to better well-being at altitude between the two Chinese subgroups are not ruled out.

GLI reference values “Other/Mixed” category tended to underestimate FEV_1_ of highlanders and to overestimate FEV_1_ of lowlanders (Tables 2, 3). The same trend was observed when using GLI “North-East Asian” category to predict FVC. Compared to other studies, our measurements of greater lung volumes at altitude are partly contradicted by data reported by Fiori in Kyrgyz and age-matched Kazakh males without excluding smokers. At 900, 2,100, and 3,200 m no altitude-related increase of lung volume was seen. Unfortunately, body height corrected data and FEV_1_ were not provided, which makes a comparison difficult (22). Mirrakhimov summarized research from the former USSR and reported that vital capacity at 3,600 m in Kyrgyz natives of the Tien Shan and Pamir mountains did not differ from corresponding values in lowlanders but no quantitative data were provided (35). Several studies in Andean (33, 36) and Himalayan highland populations found an increased FVC in highlanders. However, no study observed two random population samples of adults with the same ethnicity at high and low altitude. In Han Chinese living at 3,200–4,300 m the mean adjusted FVC values were higher than those of lowland males (by 173 ml) and females (by 136 ml) (37). Tibetans living at the same altitudes as Han Chinese show even higher differences between lowlanders and highlanders (38). Regarding FEV_1_/FVC ratio at altitude Weitz et al. reported no significant altitude-related change (37), but others postulate lower FEV_1_/FVC at higher elevation (39). Therefore, in addition to environmental conditions, genetic, socio-economic, nutritional, and other, unknown factors seem to be important. Whether the difference between highlanders and lowlanders in our sample is due to exposure to hypoxia or other environmental factors remains elusive. The same limitation applies to the interpretation of all other cited studies. As we included only Kyrgyz, the genetic difference was minimized and some kind of environmental adaption and selection can be suggested although we relied on self-declaration of ethnicity rather than on any genetic analyses. As we do not have information about migration of study participants between altitudes, length of stay and place of birth, the difference between groups may have been underestimated.

Limitations of our study include sampling in only two regions and a relatively low number of male compared to female participants which may be due to a large proportion of men working abroad and a much higher prevalence of smokers among men (62.5%) compared to women (5.9%). Thus, 283 men, compared to 33 women had to be excluded due to smoking history (Figure 1). In 2013, life expectancy in Kyrgyzstan was 67 years for men and 75 years for women (40). With our random sample strategy, we did not focus on an old population. Most notably, the number of males aged over 60 years might not be sufficient to derive reliable prediction equations as can be seen in the inspection of Figure 3.

In a mathematical point, we assume the skewness (L) to be the same as proposed by GLI and to be zero for PEF. Overall calculation of skewness for PEF was not predicted dependent on age but calculated for the sample overall and was seen to be minimal (−0.11 for males; −0.006 for females) (Figure 2). The equations for splines drawn by GLI are derived for ages 25–95 years. Including young adults aged <25 years (50 males; 68 females) did not change the results according to visual inspection suggesting that the influence of data from those subjects was minimal.

This first population based study in a representative sample of Central Asians provides predicted values for spirometry. It highlights the importance of obtaining reference equations for lung function in populations living at different altitudes. The differences in lung volumes between highlanders and lowlanders may suggest that future prediction equations incorporate a coefficient for altitude of residence. For further research we suggest investigating other ethnicities living in similar areas and at similar altitudes in order to better understand the roles of ethnicity and environment.

## Data Availability Statement

The datasets generated for this study are available on request to the corresponding author.

## Ethics Statement

The studies involving human participants were reviewed and approved by the local ethical committee in the Kyrgyzstan Health Research Authority in London (06/Q0411/97). The patients/participants provided their written informed consent to participate in this study.

## Author Contributions

All authors are responsible for the conception and design of the study, the acquisition, analysis and interpretation of data, and for drafting the work or revising it critically for important intellectual content.

### Conflict of Interest

The authors declare that the research was conducted in the absence of any commercial or financial relationships that could be construed as a potential conflict of interest.
