# Supplementary material for: Spirometry in Central Asian Lowlanders and Highlanders, a Population Based Study
Source: Front Med (Lausanne). 2020 Jan 10;6:308. doi: 10.3389/fmed.2019.00308 (PMC6966711; doi:10.3389/fmed.2019.00308)

Supplementary Material

1 Supplementary methods

1.1. Prediction equations for M and S

The prediction equations for M and S are built using the GLI prediction equation as a model. The equations for M and S have the same form, thus we address the main transformations only once.

The GLI equations for M:

$$\text{Mspline} = b_0 + b_1 \times \frac{\text{Age}}{100} + b_2 \times \left(\frac{\text{Age}}{100}\right)^2 + b_3 \times \left(\frac{\text{Age}}{100}\right)^3 + b_4 \times \left(\frac{\text{Age}}{100}\right)^4 + b_5 \times \left(\frac{\text{Age}}{100}\right)^5$$

$$M = e^{a_0 + a_1 \times \ln \text{Height} + a_1 \times \ln \text{Age} + \text{Ethnicity} + \text{Sspline}}$$

The GLI equation for S:

$$\text{Sspline} = c_0 + c_1 \times \frac{\text{Age}}{100} + c_2 \times \left(\frac{\text{Age}}{100}\right)^2 + c_3 \times \left(\frac{\text{Age}}{100}\right)^3 + c_4 \times \left(\frac{\text{Age}}{100}\right)^4 + c_5 \times \left(\frac{\text{Age}}{100}\right)^5$$

$$S = e^{p_0 + p_1 \times \ln \text{Age} + \text{Ethnicity} + \text{Sspline}}$$

In our estimation, we make some adjustments to simplify the calculation. First, the equations for the Splines are included in the regression model. Secondary, as all our measurements are Kyrgyz ethnicity, the ethnicity factor is omitted. Third, as we put the equation for M and for Mspline together, the factors a_0 and b_0 add and cannot be distinguished anymore. We assume in the following lines that a_0 and b_0 are written as a_0. The same is done for S and Sspline with p_0 and c_0 written as p_0.

The final equation for M and S which is used for multiple linear regression:

$$M = e^{a_0 + a_1 \times \ln(\text{Height}) + a_2 \times \ln(\text{Age}) + b_1 \times \ln\left(\frac{\text{Age}}{100}\right) + b_2 \times \ln\left(\frac{\text{Age}}{100}\right)^2 + b_3 \times \ln\left(\frac{\text{Age}}{100}\right)^3 + b_4 \times \ln\left(\frac{\text{Age}}{100}\right)^4 + b_5 \times \ln\left(\frac{\text{Age}}{100}\right)^5}$$

$$\ln M = a_0 + a_1 \times \ln(\text{Height}) + a_2 \times \ln(\text{Age}) + b_1 \times \ln\left(\frac{\text{Age}}{100}\right) + b_2 \times \ln\left(\frac{\text{Age}}{100}\right)^2 + b_3 \times \ln\left(\frac{\text{Age}}{100}\right)^3$$
$$+ b_4 \times \ln\left(\frac{\text{Age}}{100}\right)^4 + b_5 \times \ln\left(\frac{\text{Age}}{100}\right)^5$$

The final equation for S, which is used for multiple linear regression:

$$S = e^{p_0 + p_1 \times \ln(\text{Age}) + c_1 \times \ln\left(\frac{\text{Age}}{100}\right) + c_2 \times \ln\left(\frac{\text{Age}}{100}\right)^2 + c_3 \times \ln\left(\frac{\text{Age}}{100}\right)^3 + c_4 \times \ln\left(\frac{\text{Age}}{100}\right)^4 + c_5 \times \ln\left(\frac{\text{Age}}{100}\right)^5}$$

$$\ln S = p_0 + p_1 \times \ln(\text{Age}) + c_1 \times \ln\left(\frac{\text{Age}}{100}\right) + c_2 \times \ln\left(\frac{\text{Age}}{100}\right)^2 + c_3 \times \ln\left(\frac{\text{Age}}{100}\right)^3 + c_4 \times \ln\left(\frac{\text{Age}}{100}\right)^4 + c_5 \times \ln\left(\frac{\text{Age}}{100}\right)^5$$

When M is a measured value for either FEV_1, FVC, FEV_1/FVC or PEF, it is possible to predict factors for $a_{0\text{-}2}$ and $b_{1\text{-}5}$ for the logarithmic transformed M with regression analysis.

S is the coefficient of variation and describes the extent of standard deviation in relation to the mean[1]. The coefficient of variation needs to be calculated for each predicted M. For how to obtain S, see below.

1.2. **Calculation of S**

1.2.1. Prediction of the coefficient of variation (S)

To calculate factors for S, the coefficient of variation (S) for each age is needed. Using the equation for the corresponding ln(M), Stata calculates the standard error (S_E) of the forecast for the logarithmic transformed M (SD_ln) for each forecast. The standard error of the forecast is the standard error of the point prediction for one observation and gives us information about the accuracy of a prediction[2]. Mathematically, this is the square root of the sum of the square standard error of the regression (intrinsic risk) plus the square standard error of the mean (parameter risk)[3].

$$S_E(\text{forecast}) = \sqrt{\text{intrinsic risk}^2 + \text{parameter risk}^2} \quad [3]$$

The standard error of the forecast is the standard deviation of the error we can expect in forecasting the fitted value ($\hat{x}$) as a forecast for x. In other words, we assume the model is correct and describe the error of our prediction as the standard deviation of the error[3].

As all this values are right for the transformed variable ln(M), it is necessary to determine how the values change for non-transformed M. Applying the Delta Method, definition for Variance $Var(x) = SD^2$[4], coefficient of variation (S, $CoV = \frac{SD}{mean}$ [5] and assuming that our fitted values are the mean of subjects with the same age, height and sex in a population, we obtain the following equation for S. The modification to get this equation is described below step by step.

$$S = \sqrt{{e^{\ln M}}^2 \times \frac{SD\_ln^2}{M}}$$

$$S = SD\_ln$$

With the calculated S we can perform a regression analysis using the GLI prediction equation as a model to estimate factors for age and height.

1.2.2. Step by step modification to obtain S

How do we get SD of the non-logarithmic transformed M out of SD_ln? The solution is a mathematical method called the Delta Method [1, 6, 7]. In estimations with transformed estimates we can use the Delta Method to calculate the variance of the non-transformed variable out of the variance of the transformed variable with the help of means (mu):

$$G(x) = G(mu) + (x - mu) \times G'(mu)$$

$$Var\big(G(x)\big) = Var(x) \times G'(mu)^2$$

$$y = \ln x \;\rightarrow x = e^y$$

In our case, we want to calculate the variance of the non-logarithmic transformed M (Var) out of the variance of the logarithmic transformed M (Var_ln). Thus with Var(G(x)) = Var, Var(x) = Var_ln and G'(mu) = mean(y) the mathematical formula looks like this:

$$\text{Var} = \text{Var\_ln} \times e^{\text{mean}(y)^2}$$

The variance is defined as the square of the standard deviation (Var = $(SD)^2$ or $\sqrt{Var}$=SD). Adding this definition we obtain:

$$\text{Var} = \text{SD\_ln}^2 \times e^{\text{mean}(y)^2}$$

For the mean we take the calculated and fitted values as these values are an average value for subjects with the same characteristics (mean(y)= $\ln M$)[8].

$$\text{Var} = \text{SD\_ln}^2 \times e^{\ln M^2}$$

With this we calculate the variance of the non-transformed M. But how do we get the coefficient of variation (S) out of the variance?

The coefficient of variation is defined as standard deviation divided by the mean ($S=\frac{SD}{mean}$). As described earlier, we calculate the SD out of the variance with S= $\sqrt{Var}$. Combining the two formula:

$$S = \frac{\sqrt{Var}}{mean}$$

Inserting our variance for the non-transformed M and using M as our mean:

S = sqrt((SD_ln)^2 *exp(ln(M))^2)/M

Resulting in a value for S for each measurement, we can use our final equation and perform regression to calculate factors for p_0, p_1 and c_1 to c_5.

1.3. **The skewness (L)**

Factor L showed to be independent of ethnicity in the large population which was studied by GLI. For that reason, factors for L are not calculated but taken over from GLI for FEV_1 and FVC, FEV_1/FVC. For PEF, L is assumed to be zero.

1.4. **Final equations for the regression models**

$$M = e^{a_0 + a_1 \times \ln(\text{Height}) + a_2 \times \ln(\text{Age}) + b_1 \times \ln\left(\frac{\text{Age}}{100}\right) + b_2 \times \ln\left(\frac{\text{Age}}{100}\right)^2 + b_3 \times \ln\left(\frac{\text{Age}}{100}\right)^3 + b_4 \times \ln\left(\frac{\text{Age}}{100}\right)^4 + b_5 \times \ln\left(\frac{\text{Age}}{100}\right)^5}$$

$$S = e^{p_0 + p_1 \times \ln(\text{Age}) + c_1 \times \ln\left(\frac{\text{Age}}{100}\right) + c_2 \times \ln\left(\frac{\text{Age}}{100}\right)^2 + c_3 \times \ln\left(\frac{\text{Age}}{100}\right)^3 + c_4 \times \ln\left(\frac{\text{Age}}{100}\right)^4 + c_5 \times \ln\left(\frac{\text{Age}}{100}\right)^5}$$

$$L = q_0 + q_1 \times \ln(\text{Age}) + d_1 \times \ln\left(\frac{\text{Age}}{100}\right) + d_2 \times \ln\left(\frac{\text{Age}}{100}\right)^2 + d_3 \times \ln\left(\frac{\text{Age}}{100}\right)^3 + d_4 \times \ln\left(\frac{\text{Age}}{100}\right)^4$$
$$+ d_5 \times \ln\left(\frac{\text{Age}}{100}\right)^5 + d_6 \times \ln\left(\frac{\text{Age}}{100}\right)^6 + d_7 \times \ln\left(\frac{\text{Age}}{100}\right)^7$$

1.5. **Spirometry data processing and quality control**

Raw data were further evaluated and cleaned. As part of this cleaning some individual blows that were initially deemed acceptable by the ndd software might have been rejected on technical grounds, and in addition the Coordinating Centre in London assigned separate FEV_1, FVC, and flow quality scores in place of the single overall quality score assigned by the ndd software. These quality scores range from 0 (worst) to 4 (best). Although ATS criteria for acceptability and reproducibility have evolved over time, BOLD used the standards in effect at the time of the study and classified scores of 0-1 as unacceptable (not more than 2 acceptable tests or variability >200 ml) and scores of 2-4 as acceptable (at least 3 acceptable tests and variability ≤200 ml) [9].

2 References

1. Rice JA. Mathematical Statistics and Data Analysis. International Edition ed, 2007; p. 432.
2. STATA. Regress postestimation - Postestimation tools for regress. Stata Manuals [PDF] [cited 2018 17.01.18]; Available from: https://www.stata.com/manuals/rregresspostestimation.pdf#rregresspostestimationMethodsandformulas
3. Nau R. Review of basic staatistics and the simplest forecasting model: the sample mean. Fuqua School of Business, Duke University, 2014; pp. 5-6.
4. Kenney JF, Keeping ES. Mathematics of Statistics, Part 1. Van Nostrand, 1962.
5. Weisstein EW. Variation Coefficient From MathWorld. 2018 [cited; Available from: http://mathworld.wolfram.com/VariationCoefficient.html
6. Oehlert GW. A Note on the Delta Method. *Am Stat* 1992: 46(1): 27-29.
7. Rice JA. Mathematical Statistics and Data Analysis. International Edition ed, 2007.
8. Nau R. Review of basic staatistics and the simplest forecasting model: the sample mean. Fuqua School of Business, Duke University, 2014; p. 3.
9. Enright P, Vollmer WM, Lamprecht B, Jensen R, Jithoo A, Tan W, Studnicka M, Burney P, Gillespie S, Buist AS. Quality of spirometry tests performed by 9893 adults in 14 countries: the BOLD Study. *Respir Med* 2011: 105(10): 1507-1515.

3 Supplementary Figures and Tables

3.1 Supplementary table 1 Prediction equation for FEV_1

FEV_1 Coefficients for Females

	M		S		L	
Intercept	a_0	-18.01565	p_0	2.744162	q_0	1.1540
Height	a_1	1.671706				
Age	a_2	7.74713	p_1	-3.325563	q_1	0
	**Mspline**		**Sspline**		**Lspline**	
	b_1	-98.2454	c_1	40.00436	d_1	0
	b_2	241.5144	c_2	-93.70416	d_2	0
	b_3	-385.6172	c_3	142.5323	d_3	0
	b_4	330.9596	c_4	-119.1068	d_4	0
	b_5	-115.6229	c_5	41.66702	d_5	0
					d_6	0
					d_7	0

FEV_1 Coefficients for Males

	M		S		L	
Intercept	a_0	12.07667	p_0	4.815081	q_0	0.8866
Height	a_1	2.069277				
Age	a_2	-16.27688	p_1	-4.600079	q_1	0.0850
	**Mspline**		**Sspline**		**Lspline**	
	b_1	219.6743	c_1	54.50138	d_1	0
	b_2	-566.8287	c_2	-124.8397	d_2	0
	b_3	924.7084	c_3	185.7966	d_3	0
	b_4	-807.5969	c_4	-153.3491	d_4	0
	b_5	286.2138	c_5	53.99652	d_5	0
					d_6	0
					d_7	0

$$M = e^{a_0 + a_1 \times \ln(\text{Height}) + a_2 \times \ln(\text{Age}) + b_1 \times \ln\left(\frac{\text{Age}}{100}\right) + b_2 \times \ln\left(\frac{\text{Age}}{100}\right)^2 + b_3 \times \ln\left(\frac{\text{Age}}{100}\right)^3 + b_4 \times \ln\left(\frac{\text{Age}}{100}\right)^4 + b_5 \times \ln\left(\frac{\text{Age}}{100}\right)^5}$$

$$S = e^{p_0 + p_1 \times \ln(\text{Age}) + c_1 \times \ln\left(\frac{\text{Age}}{100}\right) + c_2 \times \ln\left(\frac{\text{Age}}{100}\right)^2 + c_3 \times \ln\left(\frac{\text{Age}}{100}\right)^3 + c_4 \times \ln\left(\frac{\text{Age}}{100}\right)^4 + c_5 \times \ln\left(\frac{\text{Age}}{100}\right)^5}$$

$$L = q_0 + q_1 \times \ln(\text{Age}) + d_1 \times \ln\left(\frac{\text{Age}}{100}\right) + d_2 \times \ln\left(\frac{\text{Age}}{100}\right)^2 + d_3 \times \ln\left(\frac{\text{Age}}{100}\right)^3 + d_4 \times \ln\left(\frac{\text{Age}}{100}\right)^4 + d_5 \times \ln\left(\frac{\text{Age}}{100}\right)^5 + d_6 \times \ln\left(\frac{\text{Age}}{100}\right)^6 + d_7 \times \ln\left(\frac{\text{Age}}{100}\right)^7$$

3.2 Supplementary table 2 Prediction equation for FVC

FVC Coefficients for Females

	M		S		L	
Intercept	a_0	-14.58863	p_0	2.672409	q_0	0.8236
Height	a_1	1.795792				
Age	a_2	4.636313	p_1	-3.325566	q_1	0

	Mspline		Sspline		Lspline	
	b_1	-55.27524	c_1	40.00441	d_1	0
	b_2	133.034	c_2	-93.70426	d_2	0
	b_3	-213.2442	c_3	142.5325	d_3	0
	b_4	185.2339	c_4	-119.1069	d_4	0
	b_5	-65.59613	c_5	41.66705	d_5	0
					d_6	0
					d_7	0

FVC Coefficients for Males

	M		S		L	
Intercept	a_0	8.486290	p_0	4.569424	q_0	0.9481
Height	a_1	2.104119				
Age	a_2	-13.15918	p_1	-4.600081	q_1	0

	Mspline		Sspline		Lspline	
	b_1	169.985	c_1	54.5014	d_1	0
	b_2	-417.9367	c_2	-124.8398	d_2	0
	b_3	654.1605	c_3	185.7966	d_3	0
	b_4	-553.6821	c_4	-153.3491	d_4	0
	b_5	192.0865	c_5	53.99654	d_5	0
					d_6	0
					d_7	0

$$M = e^{a_0 + a_1 \times \ln(\text{Height}) + a_2 \times \ln(\text{Age}) + b_1 \times \ln\left(\frac{\text{Age}}{100}\right) + b_2 \times \ln\left(\frac{\text{Age}}{100}\right)^2 + b_3 \times \ln\left(\frac{\text{Age}}{100}\right)^3 + b_4 \times \ln\left(\frac{\text{Age}}{100}\right)^4 + b_5 \times \ln\left(\frac{\text{Age}}{100}\right)^5}$$

$$S = e^{p_0 + p_1 \times \ln(\text{Age}) + c_1 \times \ln\left(\frac{\text{Age}}{100}\right) + c_2 \times \ln\left(\frac{\text{Age}}{100}\right)^2 + c_3 \times \ln\left(\frac{\text{Age}}{100}\right)^3 + c_4 \times \ln\left(\frac{\text{Age}}{100}\right)^4 + c_5 \times \ln\left(\frac{\text{Age}}{100}\right)^5}$$

$$L = q_0 + q_1 \times \ln(\text{Age}) + d_1 \times \ln\left(\frac{\text{Age}}{100}\right) + d_2 \times \ln\left(\frac{\text{Age}}{100}\right)^2 + d_3 \times \ln\left(\frac{\text{Age}}{100}\right)^3 + d_4 \times \ln\left(\frac{\text{Age}}{100}\right)^4 + d_5 \times \ln\left(\frac{\text{Age}}{100}\right)^5 + d_6 \times \ln\left(\frac{\text{Age}}{100}\right)^6 + d_7 \times \ln\left(\frac{\text{Age}}{100}\right)^7$$

3.3 Supplementary table 3 Prediction equation for FEV_1/FVC

FEV_1/FVC Coefficients for Females

	M		S		L	
Intercept	a_0	-3.427023	p_0	2.037301	q_0	7.032
Height	a_1	-.1240862				
Age	a_2	3.11082	p_1	-3.325566	q_1	-1.197

	Mspline		Sspline		Lspline	
	b_1	-42.97019	c_1	40.0044	d_1	0
	b_2	108.4805	c_2	-93.70422	d_2	0
	b_3	-172.3731	c_3	142.5324	d_3	0
	b_4	145.7258	c_4	-119.1069	d_4	0
	b_5	-50.02682	c_5	41.66702	d_5	0
					d_6	0
					d_7	0

FEV_1/FVC Coefficients for Males

	M		S		L	
Intercept	a_0	3.590386	p_0	-2.349685	q_0	4.7101
Height	a_1	-.0348413				
Age	a_2	-3.117703	p_1	-1.31443	q_1	-0.6774

	Mspline		Sspline		Lspline	
					d_0	1.708
	b_1	49.68931	c_1	19.79478	d_1	-39.328
	b_2	-148.8921	c_2	-61.02065	d_2	287.392
	b_3	270.5481	c_3	117.9693	d_3	-980.699
	b_4	-253.9150	c_4	-118.3113	d_4	1820.089
	b_5	94.12736	c_5	48.2295	d_5	-1909.653
					d_6	1068.022
					d_7	-247.984

$$M = e^{a_0 + a_1 \times \ln(\text{Height}) + a_2 \times \ln(\text{Age}) + b_1 \times \ln\left(\frac{\text{Age}}{100}\right) + b_2 \times \ln\left(\frac{\text{Age}}{100}\right)^2 + b_3 \times \ln\left(\frac{\text{Age}}{100}\right)^3 + b_4 \times \ln\left(\frac{\text{Age}}{100}\right)^4 + b_5 \times \ln\left(\frac{\text{Age}}{100}\right)^5}$$

$$S = e^{p_0 + p_1 \times \ln(\text{Age}) + c_1 \times \ln\left(\frac{\text{Age}}{100}\right) + c_2 \times \ln\left(\frac{\text{Age}}{100}\right)^2 + c_3 \times \ln\left(\frac{\text{Age}}{100}\right)^3 + c_4 \times \ln\left(\frac{\text{Age}}{100}\right)^4 + c_5 \times \ln\left(\frac{\text{Age}}{100}\right)^5}$$

$$L = q_0 + q_1 \times \ln(\text{Age}) + d_1 \times \ln\left(\frac{\text{Age}}{100}\right) + d_2 \times \ln\left(\frac{\text{Age}}{100}\right)^2 + d_3 \times \ln\left(\frac{\text{Age}}{100}\right)^3 + d_4 \times \ln\left(\frac{\text{Age}}{100}\right)^4 + d_5 \times \ln\left(\frac{\text{Age}}{100}\right)^5 + d_6 \times \ln\left(\frac{\text{Age}}{100}\right)^6 + d_7 \times \ln\left(\frac{\text{Age}}{100}\right)^7$$

3.4 Supplementary table 4 Prediction equation for PEF

PEF Coefficients for Females

	M		S	
Intercept	a_0	-3.742475	p_0	3.081919
Height	a_1	1.231473		
Age	a_2	0.1209878	p_1	-3.330493

	Mspline		Sspline	
	b_1	-9.457709	c_1	39.81217
	b_2	37.06981	c_2	-92.73887
	b_3	-73.54428	c_3	140.4142
	b_4	68.74396	c_4	-116.9282
	b_5	-24.84668	c_5	40.82052

PEF Coefficients for Males

	M		S	
Intercept	a_0	-24.93162	p_0	5.02263
Height	a_1	1.271406		
Age	a_2	12.69888	p_1	-4.662469

	Mspline		Sspline	
	b_1	-117.4898	c_1	55.48152
	b_2	173.4365	c_2	-127.675
	b_3	-96.31546	c_3	190.8528
	b_4	-57.42041	c_4	-158.0645
	b_5	62.46204	c_5	55.75321

$$M = e^{a_0 + a_1 \times \ln(\text{Height}) + a_2 \times \ln(\text{Age}) + b_1 \times \ln\left(\frac{\text{Age}}{100}\right) + b_2 \times \ln\left(\frac{\text{Age}}{100}\right)^2 + b_3 \times \ln\left(\frac{\text{Age}}{100}\right)^3 + b_4 \times \ln\left(\frac{\text{Age}}{100}\right)^4 + b_5 \times \ln\left(\frac{\text{Age}}{100}\right)^5}$$

$$S = e^{p_0 + p_1 \times \ln(\text{Age}) + c_1 \times \ln\left(\frac{\text{Age}}{100}\right) + c_2 \times \ln\left(\frac{\text{Age}}{100}\right)^2 + c_3 \times \ln\left(\frac{\text{Age}}{100}\right)^3 + c_4 \times \ln\left(\frac{\text{Age}}{100}\right)^4 + c_5 \times \ln\left(\frac{\text{Age}}{100}\right)^5}$$

L = 0

3.5 Supplementary figure 1 Patient flow lowlanders (Chui region)

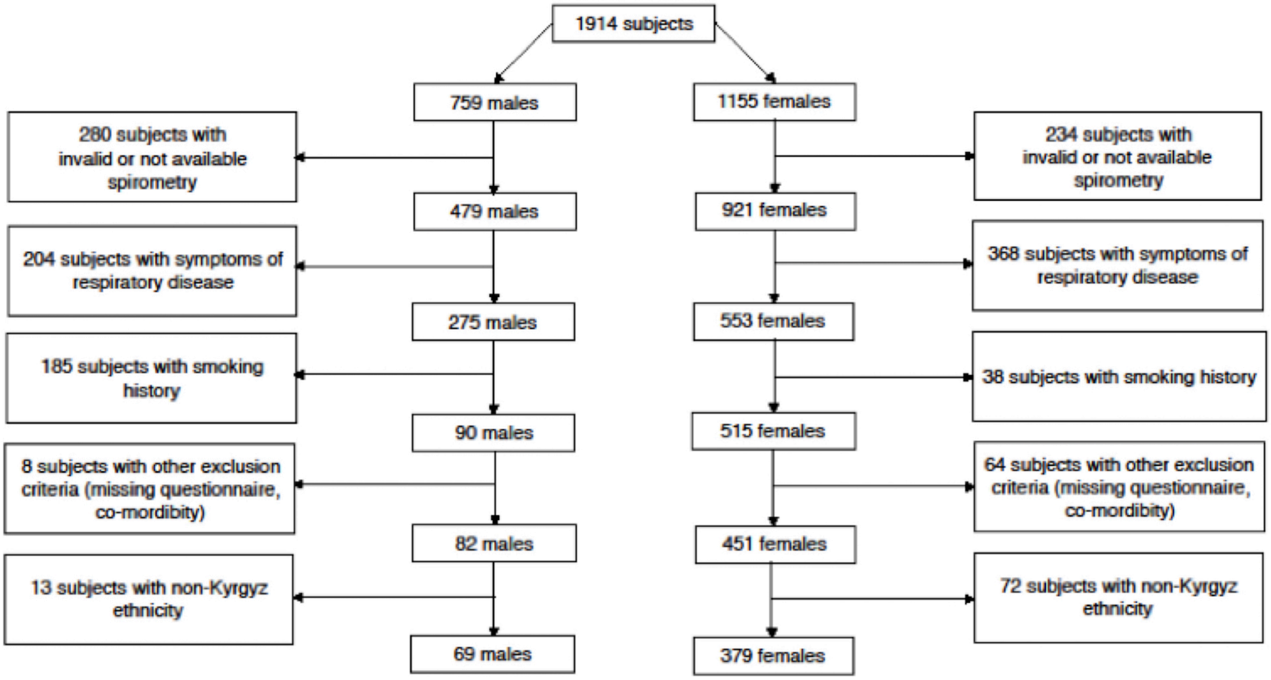

3.6 Supplementary figure 2 Patient flow highlanders (Naryn region)

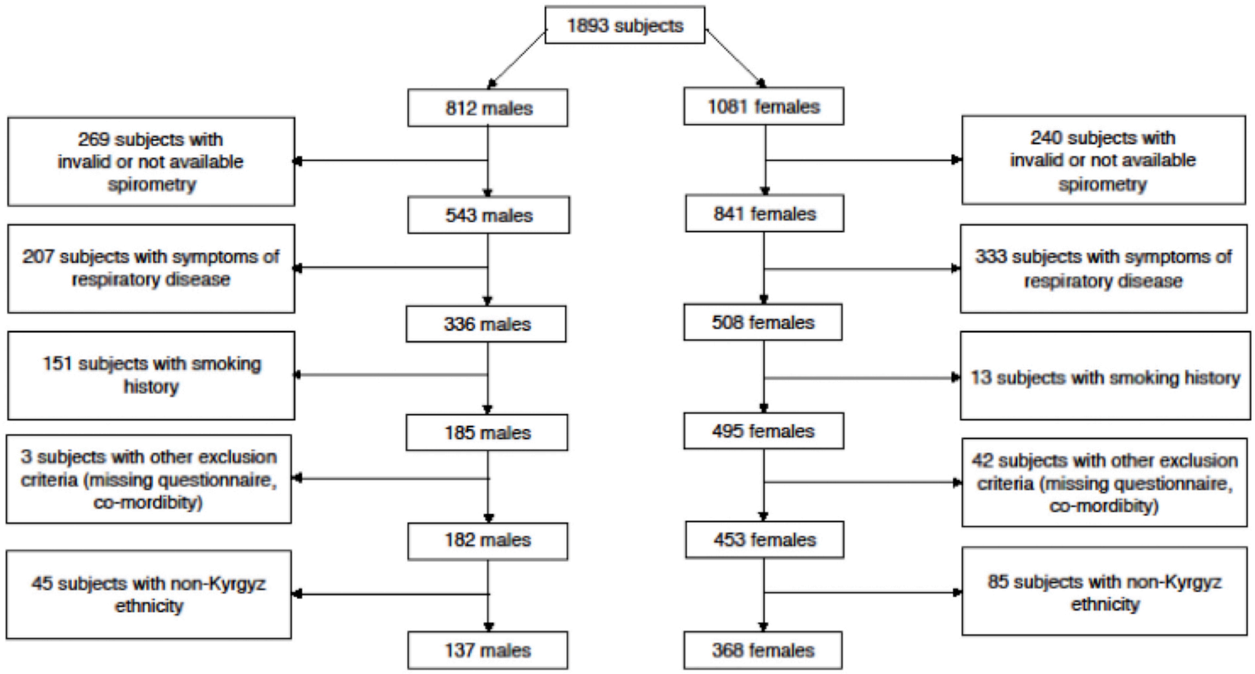

3.7 Supplementary figure 3 Distribution of age, height and weight

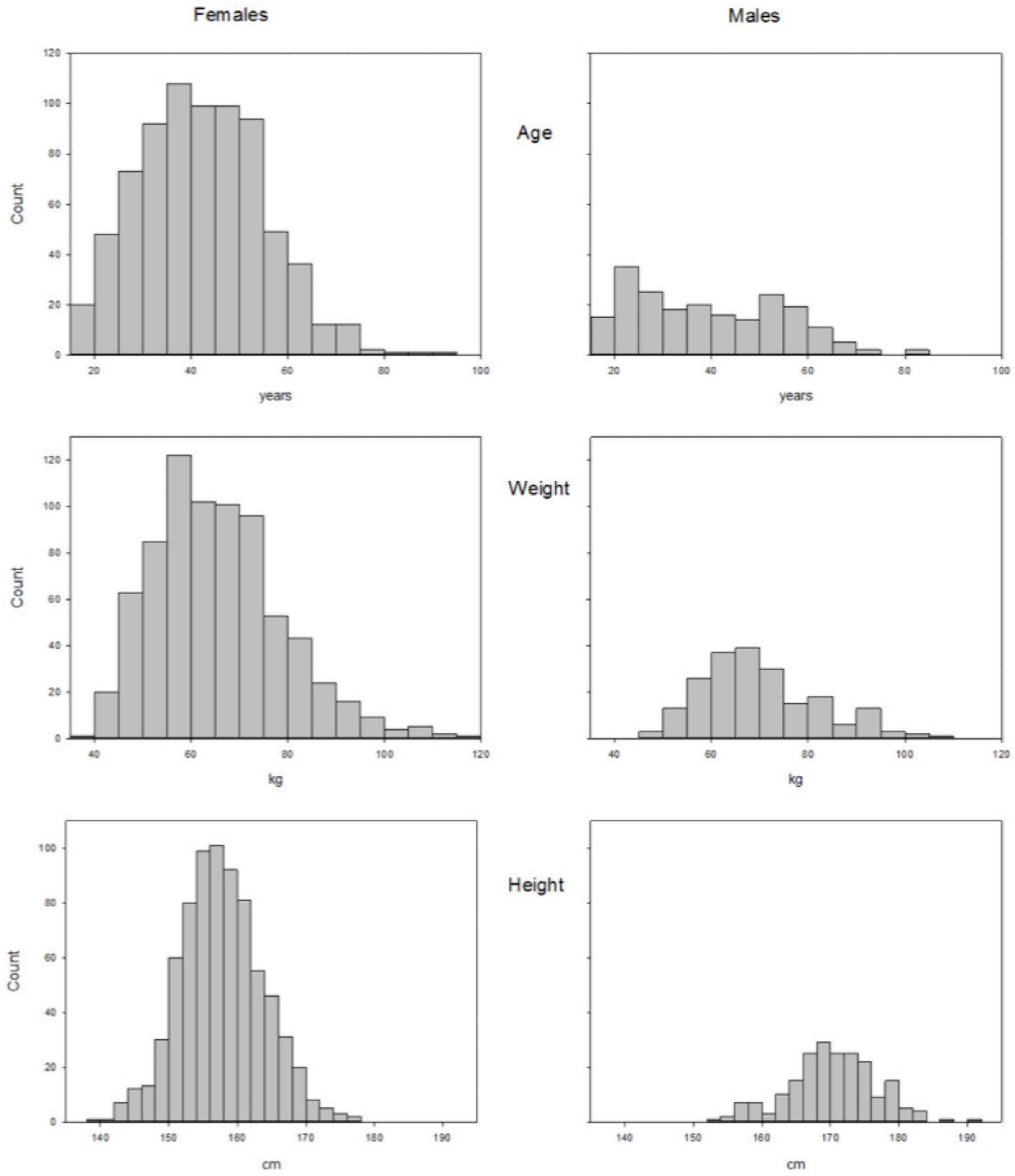

Supplement: Supplementary file 1 [file Data_Sheet_1.PDF]
